# Idiopathic Thrombocytopenic Purpura: A Rare Syndrome with Alemtuzumab, Review of Monitoring Protocol

**DOI:** 10.7759/cureus.5715

**Published:** 2019-09-20

**Authors:** Deepika Sarvepalli, Mamoon Ur Rashid, Waqas Ullah, Yousaf Zafar, Muzammil Khan

**Affiliations:** 1 Internal Medicine, Guntur Medical College, Guntur, IND; 2 Internal Medicine, AdventHealth, Orlando, USA; 3 Internal Medicine, Abington Hospital - Jefferson Health, Abington, USA; 4 Internal Medicine, University of Missouri - Kansas City School of Medicine, Kansas City, USA; 5 Internal Medicine, Khyber Teaching Hospital, Peshawar, PAK

**Keywords:** alemtuzumab, itp, multiple sclerosis, alemtuzumab autoimmunity, platelet disorders

## Abstract

Alemtuzumab, a humanized monoclonal antibody that targets surface molecule CD52, causes rapid and complete depletion of circulating T- and B-lymphocytes through antibody-dependent cell-mediated and complement-mediated cytotoxicity. Alemtuzumab has demonstrated superior efficacy compared to subcutaneous interferon beta-1a (SC IFNB-1a) in patients with multiple sclerosis (MS). Alemtuzumab treatment causes a rare and distinct form of secondary immune thrombocytopenic purpura (ITP), characterized by delayed onset, responsiveness to conventional therapies, and prolonged remission following treatment. In phase two and three clinical trials, the incidence of ITP was higher with alemtuzumab treatment compared to the patients receiving SC IFNB-1a. Here we report a case of ITP occurring two years after the first treatment with alemtuzumab. The patient recovered completely after a timely diagnosis and adequate treatment. Rigorous patient education and careful complete blood count (CBC) monitoring by the physician are critical for early identification and treatment of this potentially fatal disorder.

## Introduction

Alemtuzumab is a humanized monoclonal antibody that targets surface molecule CD52, expressed at high levels on T- and B-lymphocytes, and at very low levels on natural killer cells, monocytes, and macrophages. Alemtuzumab causes rapid and complete depletion of circulating T- and B-lymphocytes through antibody-dependent cell-mediated and complement-mediated cytotoxicity [1].

In November 2014, alemtuzumab (Lemtrada®, Sanofi Genzyme, Cambridge, MA, USA) was approved by the Food and Drug Administration (FDA) for the treatment of active relapsing remitting multiple sclerosis (RRMS). Alemtuzumab has demonstrated superior efficacy (low relapse rates, improved disability, and decreased brain volume loss) compared to subcutaneous interferon beta-1a (SC IFNB-1a) in patients with RRMS [2-3].

Common adverse effects (AEs) of alemtuzumab treatment include infections, infusion reactions, and secondary autoimmune reactions [4-6]. Autoimmune AEs consisted of thyroid disorders (39% in CARE-MS six year follow-up study), immune thrombocytopenic purpura (ITP) (2.6% in CARE-MS six year follow-up study), and autoimmune renal disease (0.2% in CARE-MS six year follow-up study) that occurred years after first treatment [6].

Immune thrombocytopenic purpura is a bleeding disorder characterized by isolated low platelet count not associated with a systemic disease. It can be primary (no predisposing agent) or secondary to a predisposing factor such as drugs, infections, autoimmune disorders, or neoplasms. Alemtuzumab treatment causes a distinct form of secondary ITP, characterized by delayed onset, responsiveness to conventional therapies, and prolonged remission following treatment [7].

Here we report a case of ITP occurring two years after the first treatment with alemtuzumab.

## Case presentation

A 45-year-old female with a history of multiple sclerosis (MS) was admitted to the hospital for low platelet count, after being referred by her primary care physician (PCP). She was placed on alemtuzumab as a part of the treatment for MS. Treatment was initiated two years ago, and she received two doses one year apart, with the last dose administered one year ago. The patient was continuously monitored with monthly complete blood count (CBC). On physical examination, the patient had mucocutaneous manifestations of thrombocytopenia; petechia on the chest and bruises on upper limbs were noticed. The patient did not have a history of Wiskott-Aldrich syndrome, thrombocytopenia - absent radius syndrome, May-Hegglan anomaly, gray platelet syndrome, Upshaw-Shulman syndrome, or Bernard-Soulier syndrome. On the first day of admission, the patient had a platelet count of only 3000/μL. Her medical records from the last three months showed a serial decline of platelet count.

Further investigations were done on the patient, in search for the etiology of thrombocytopenia. The patient did not have any signs of thrombotic thrombocytopenic purpura (TTP), hemolytic uremic syndrome (HUS), and disseminated intravascular coagulation (DIC) as evidenced by the absence of schistocytes on peripheral smear, normal lactate dehydrogenase (LDH), and normal renal functions and normal prothrombin time/activated partial thromboplastin time (PT /aPTT). Moreover, patient‘s home medications were reviewed, including ampura, oxybutynin, donepezil, baclofen which were not associated with thrombocytopenia. Other labs such as vitamin B12 level, thyroid stimulating hormone (TSH), antinuclear antibody (ANA), rheumatoid factor (RF), human immunodeficiency virus (HIV), Ebstein-Barr virus (EBV), hepatitis serology were unremarkable. After excluding other causes, the patient was treated for ITP secondary to alemtuzumab, with prednisone 1 mg/kg. Soon, the patient showed a good response to treatment; platelet count increased, and bruises and petechiae resolved within a few days.

## Discussion

The ITP syndrome is recognized by antibody and cell-mediated platelet destruction and suppression of platelet production, which may occur without any predisposing factor (primary ITP) or secondary due to conditions, including autoimmune disorders, neoplasms, congenital immune deficiencies, drugs, and infections. The diagnostic criteria of ITP, outlined by an international working group, include either a confirmed platelet count of ≥50 × 109/L and <100 × 109/L on ≥ two consecutive occasions over one month, or a confirmed platelet count <50 × 109/L on ≥ two consecutive occasions over any period of time; and normal hemoglobin (Hb), normal white blood cell (WBC) count, no splenomegaly, normal peripheral smear except for low platelets, and no evidence of an alternative nonautoimmune etiology of thrombocytopenia [8]. In our case, ITP was thought to be secondary to alemtuzumab treatment as no other etiology was found, and a normal platelet count was documented before starting the medication.

The incidence of ITP in patients treated with alemtuzumab is higher compared to the general population and patients receiving SC IFNB-1a. During the CAMMS223 phase II clinical trial [6], 334 patients were tested for the development of ITP after therapy. Patients were randomly assigned to either alemtuzumab or SC IFNB-1a and followed up for 4.5 years. While ITP was observed in 2.8% (six out of 216) of the people treated with alemtuzumab, it was noticed in only 0.9%(one out of 107) of the patients on SC IFNB-1a [6]. Moreover, the occurrence of ITP with alemtuzumab was dose-dependent (1.9% in 12 mg/d vs. 3.7% in 24 mg/d). The overall incidence rate of ITP in the three cohorts: alemtuzumab 12 mg/d, alemtuzumab 24 mg/d, and SC IFNB-1a were 4.2, 8.0, and 2.7 per 1000 person-years, respectively [6].

Alemtuzumab-induced ITP is distinct from both drug-induced ITP (DITP) and adult primary ITP in terms of the onset and course of the disease. DITP typically occurs within days of exposure to the drug and requires the presence of the drug in the blood. DITP usually resolves within days after discontinuing the drug. In contrast, alemtuzumab-associated ITP appears in a delayed fashion and can occur months to years after the last dose of the medication [7]. A point worth mentioning here is the unique dosing schedule of alemtuzumab in the treatment of RRMS. The recommended schedule comprises intravenous infusion of 12 mg of alemtuzumab on each of five consecutive days (total dose of 60 mg) and on each of three consecutive days (36 mg total dose) 12 months later. During the clinical development program, alemtuzumab-induced ITP occurred at a median time of 10.5 months after the last dose, and all the cases happened within 48 months after the last infusion of the medication [9-10]. This delayed pattern of alemtuzumab autoimmunity strikes a resemblance with autoimmune disorders occurring in the setting of antiretroviral therapy in HIV-infected patients and autologous hematopoietic stem cell transplantation, suggesting a likely disorder of lymphocyte repopulation [11-14].

In adults, primary ITP typically runs a chronic course, and only 10% of the patients experience durable remission after front-line treatment, comprising corticosteroids, intravenous immunoglobulin (IVIg), anti-RhD immunoglobulin (anti-D), and/or platelet transfusion [15]. It often requires continuous monitoring and subsequent treatment. In contradistinction, alemtuzumab-associated ITP is self-limiting and achieves lasting remission in 70%-80% of cases with the front-line treatment alone while remaining few cases may require rituximab, the second-line drug. In this regard, it resembles childhood ITP that assumes a self-limiting course as well and results in spontaneous recovery within weeks to months [16].

In 2018, Cuker et al. [17] presented an analysis of 1485 alemtuzumab-treated MS patients, based on the data pooled from the phase two and three studies, and the extension study, spanning from December 2002 to the end of February 2016. The overall incidence of ITP was 2.3% (n = 34), out of which, 24 patients received 12 mg of alemtuzumab, and nine received 24 mg, after a median follow-up of 6.1 years. Unfortunately, one person died from life-threatening ITP (diagnosed postmortem) soon after the study's inception, and this called for the introduction of strict risk monitoring program in all the remaining patients. Out of the 33 patients diagnosed with the protocol-defined ITP, 22 patients recovered with first-line therapy (IVIg, corticosteroids, and/or platelet transfusion), seven had a good response to second-line therapy (rituximab or splenectomy), and two had spontaneous resolution. Interestingly, all the patients achieved sustained remission with treatment, and no deaths were reported after the index case. The results emphasize the importance of patient education and thorough clinical monitoring in the early detection and effective management of ITP.

**Figure 1:**
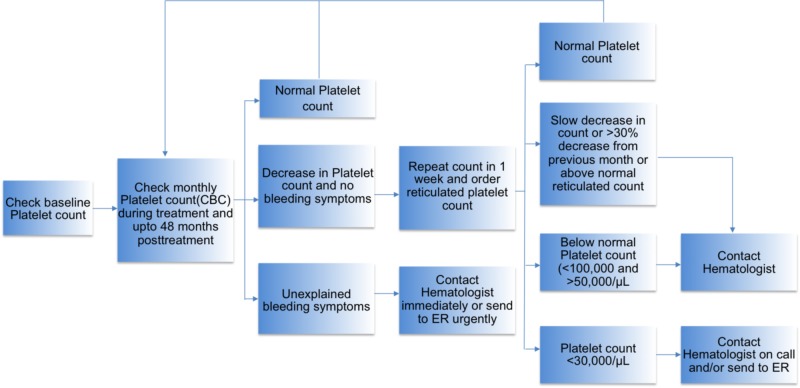
Algorithm for recommendations in monitoring of platelet counts in patients treated with alemtuzumab. CBC, complete blood count; ER, emergency room. Normal platelet count is 150,000-450,000 platelets/μL of blood.

**Figure 2:**
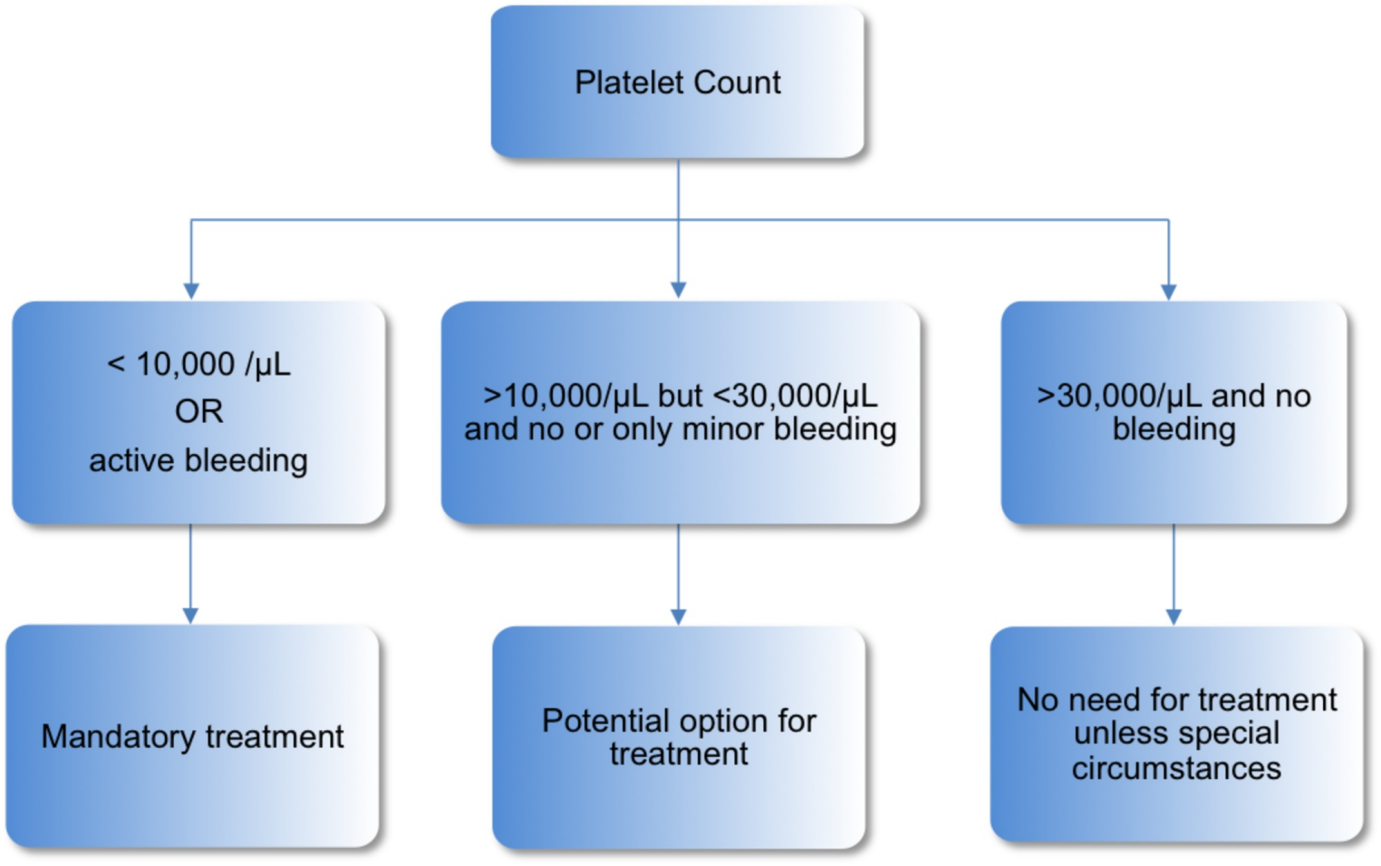
Algorithm for initiation of treatment in ITP. ITP, idiopathic thrombocytopenic purpura.

In our case, the patient received her last dose of alemtuzumab 12 months before developing ITP. Since the beginning of the treatment, the patient was regularly followed with monthly CBC monitoring. The algorithm for monitoring of patients on alemtuzumab is shown in Figure 1. As soon as the patient developed petechiae and bruises, she was admitted to the hospital for further management and the diagnosis of alemtuzumab-induced ITP was confirmed. Soon, the patient was started on guideline-based therapy, as outlined in Figure 2. She received corticosteroids and platelet transfusion, and subsequently, her platelet count improved.

Few theories were postulated to explain the mechanism of ITP in alemtuzumab-treated patients. One of the hypotheses is that alemtuzumab causes selective depletion and predominant proliferation of T cells, driven by high serum levels of interleukin -21 (IL -21) [18]. Another hypothesis is that autoimmunity may arise from rapid repopulation of B-lymphocytes, which was observed mostly in people with a genetic predisposition for autoimmunity [19]. Both of the hypotheses point to defects during lymphocyte reconstitution. Apart from these, a family history of autoimmune disease and smoking are found to be independent risk factors for alemtuzumab autoimmunity [20]. A comprehensive understanding of the mechanism of alemtuzumab-associated ITP is needed at this juncture. Serial testing of patients for the identification of additional immune markers in ITP and immune reconstitution at various phases including, acute phase of ITP, baseline and in the remission phase may elucidate the pathogenesis of ITP in general, and alemtuzumab-associated ITP, in detail. Detection of additional predictive biomarkers might facilitate early identification of potential patients at risk of developing ITP. Meanwhile, rigorous patient education and careful CBC monitoring by the physician are critical to early identification and treatment of the disorder.

## Conclusions

In conclusion, a rare and distinct form of secondary ITP has been associated with alemtuzumab, with good response to front-line ITP treatment agents and delayed presentation after the medication use. Investigations are currently under study to understand the possible mechanism of the syndrome, and further surveillance and management. In the meantime, careful monitoring of CBC by the physician, coupled with patient education for early identification of ITP signs and symptoms can aid in a timely diagnosis of this potentially fatal syndrome.

## References

[1] Havrdova E, Horakova D, Kovarova I (2015). Alemtuzumab in the treatment of multiple sclerosis: key clinical trial results and considerations for use. Ther Adv Neurol Disord.

[2] Cohen JA, Coles AJ, Arnold DL (2012). Alemtuzumab versus interferon beta 1a as first-line treatment for patients with relapsing-remitting multiple sclerosis: a randomised controlled phase 3 trial. Lancet.

[3] Coles AJ, Twyman CL, Arnold DL (2012). Alemtuzumab for patients with relapsing multiple sclerosis after disease-modifying therapy: a randomised controlled phase 3 trial. Lancet.

[4] Ziemssen T, Thomas K (2017). Alemtuzumab in the long-term treatment of relapsing-remitting multiple sclerosis: an update on the clinical trial evidence and data from the real world. Ther Adv Neurol Disord.

[5] Coles AJ, Boyko AN, Cohen JA (2019). Alemtuzumab provides durable improvements in clinical outcomes in treatment-naive patients with active relapsing-remitting multiple sclerosis over 6 years in the absence of continuous treatment (CARE-MS I). Mult Scler.

[6] Fox E, Alroughani R, Brassat D (2016). Efficacy of alemtuzumab is durable over 6 years in patients with active relapsing-remitting multiple sclerosis and an inadequate response to prior therapy in the absence of continuous treatment (CARE-MS II). Mult Scler.

[7] Cuker A, Coles AJ, Sullivan H (2011). A distinctive form of immune thrombocytopenia in a phase 2 study of alemtuzumab for the treatment of relapsing-remitting multiple sclerosis. Blood.

[8] Rodeghiero F, Stasi R, Gernsheimer T (2009). Standardization of terminology, definitions and outcome criteria in immune thrombocytopenic purpura of adults and children: report from an international working group. Blood.

[9] (2019). Lemtrada (Alemtuzumab) summary of product characteristics. https://www.ema.europa.eu/en/documents/product-information/lemtrada-epar-product-information_en.pdf.

[10] (2019). Lemtrada (Alemtuzumab) prescribing information. Cambridge, MA: Genzyme Corporation.

[11] Chen F, Day SL, Metcalfe RA (2005). Characteristics of autoimmune thyroid disease occurring as a late complication of immune reconstitution in patients with advanced human immunodeficiency virus (HIV) disease. Medicine.

[12] Jubault V, Penfornis A, Schillo F (2000). Sequential occurrence of thyroid autoantibodies and graves’ disease after immune restoration in severely immunocompromised human immunodeficiency virus-1-infected patients. J Clin Endocrinol Metab.

[13] Hequet O, Salles G, Ketterer N (2003). Autoimmune thrombocytopenic purpura after autologous stem cell transplantation. Bone Marrow Transplant.

[14] Jillella AP, Kallab AM, Kutlar A (2000). Autoimmune thrombocytopenia following autologous hematopoietic cell transplantation: review of literature and treatment options. Bone Marrow Transplant.

[15] Pamuk G, Pamuk O, Baslar Z (2002). Overview of 321 patients with idiopathic thrombocytopenic purpura. Ann Hematol.

[16] Zeller B, Rajantie J, Hedlund‐Treutiger I (2005). Childhood idiopathic thrombocytopenic purpura in the Nordic countries: epidemiology and predictors of chronic disease. Acta Paediatr.

[17] Cuker A, Bass AD, Nadj C (2019). Immune thrombocytopenia in alemtuzumab-treated MS patients: incidence, detection, and management. Mult Scler.

[18] Jones J L, Thompson S A, Loh P (2013). Human autoimmunity after lymphocyte depletion is caused by homeostatic T-cell proliferation. Proc Natl Acad Sci USA.

[19] Baker D, Herrod SS, Alvarez-Gonzalez C, Giovannoni G, Schmierer K (2017). Interpreting lymphocyte reconstitution data from the pivotal phase 3 trials of alemtuzumab. JAMA Neurol.

[20] Cossburn M, Pace A A, Jones J (2011). Autoimmune disease after alemtuzumab treatment for multiple sclerosis in a multicenter cohort. Neurology.

